# Role of Salivary MicroRNA and Cytokines in the Diagnosis and Prognosis of Oral Squamous Cell Carcinoma

**DOI:** 10.3390/ijms222212215

**Published:** 2021-11-11

**Authors:** Francisco Javier Manzano-Moreno, Victor J. Costela-Ruiz, Enrique García-Recio, Maria Victoria Olmedo-Gaya, Concepción Ruiz, Candelaria Reyes-Botella

**Affiliations:** 1 Biomedical Group (BIO277), Department of Stomatology, School of Dentistry, University of Granada, 18071 Granada, Spain; fjmanza@ugr.es (F.J.M.-M.); creyes@ugr.es (C.R.-B.); 2 Instituto Investigación Biosanitaria, ibs.Granada, 18071 Granada, Spain; vircoss@ugr.es (V.J.C.-R.); egr@ugr.es (E.G.-R.); 3 Biomedical Group (BIO277), Department of Nursing, Faculty of Health Sciences, Campus de Ceuta, University of Granada, 51001 Ceuta, Spain; 4 Biomedical Group (BIO277), Department of Nursing, Faculty of Health Sciences, Campus de Melilla, University of Granada, 52005 Melilla, Spain; 5 Department of Stomatology, School of Dentistry, University of Granada, 18071 Granada, Spain; mvolmedo@ugr.es; 6 Biomedical Group (BIO277), Department of Nursing, Faculty of Health Sciences, University of Granada, 18016 Granada, Spain; 7 Institute of Neuroscience, University of Granada, 18071 Granada, Spain

**Keywords:** salivary biomarker, microRNA, cytokines, oral pathology, diagnosis, oral cancer

## Abstract

Oral squamous cell carcinoma (OSCC) is the most prevalent oral malignant tumor worldwide. An early diagnosis can have a major positive impact on its prognosis. Human saliva contains cytokines, DNA and RNA molecules, circulating cells, and derivatives of tissues and extracellular vesicles, among other factors that can serve as biomarkers. Hence, the analysis of saliva may provide useful information for the early diagnosis of OSCC for its prognosis. The objective of this review was to determine the potential usefulness of salivary biomarkers (cytokines and microRNA) to diagnose OSCC and improve its prognosis. A combination of salivary miRNA and proteomic data could allow a definitive and early diagnosis to be obtained. However, there remains a need to optimize and standardize the protocols used to quantify miRNAs.

## 1. Introduction

Oral squamous cell carcinoma (OSCC) is the most prevalent oral malignant tumor worldwide, with a five-year survival rate of only 50%. An early diagnosis by primary care physicians or odontologists can have a major positive impact on its prognosis. However, the absence of symptoms and the lack of awareness by patients of the risk factors (tobacco, betel quid and alcohol) mean that the diagnosis is often made at a late stage, worsening the prognosis and increasing the mortality rate [1,2,3].

The diagnostic method of choice is to take a biopsy for pathological study, but this is sometimes not ordered because of its invasive nature, a lack of appropriate professional training, or an inability to meet the economic cost. Brush biopsy or toluidine blue staining can provide an early diagnosis but are known to miss lesions in localizations of difficult access, limiting their reliability. Detection of OSCC by conventional cancer screening strategies is also hampered by the heterogeneity between and within tumors and by their dynamic behavior, with changes in their molecular profile over time and in response to treatment. More effective techniques are therefore needed in order to deliver an early and reliable diagnosis [4]. Molecular techniques have attracted increasing attention, with reports that they can offer an early and accurate diagnosis, increased therapeutic success, and a reduced recurrence rate [5].

Human saliva contains cytokines, DNA and RNA molecules, circulating cells, and derivatives of tissues and extracellular vesicles (EVs), among other factors that can serve as biomarkers. Hence, the analysis of saliva may provide useful information for the early diagnosis of OSCC for its prognosis [1]. The objective of this review was to determine the potential usefulness of salivary biomarkers (cytokines and microRNA) to diagnose OSCC and improve its prognosis.

## 2. Salivary MicroRNA (miRNA) in OSCC Diagnosis and Prognosis

MiRNAs are small single-chain RNA molecules (19–21 nucleotides) transcribed by cell polymerase RNA and subjected to a double sequential cut in the nucleus (primary miRNA) and cytoplasm (precursor miRNA) (Table 1). Therefore, depending on their level of complementarity with the target, they can inhibit messenger RNA (mRNA) or its translation [1,6,7].

MiRNAs can be released in body fluids as cell-free miRNAs associated with RNA binding proteins or selectively packed in extracellular vesicles [27]. MiRNAs are involved in regulation of various biological processes, such as cell differentiation, proliferation, apoptosis and in embryonic and tissue development. At the level of bone metabolism, various miRNAs are emerging that are involved in their regulation [28]. Their expression is not modified in body tissues or fluids and is frequently tumor-specific. They are also the main regulators of gene expression and are therefore vitally important to detect incipient malignant transformation. Accordingly, the analysis of miRNA profiles in patients with cancer represents a new paradigm in the development of biomarkers for the clinical diagnosis of this disease.

Furthermore, the expression of certain miRNAs in the tumor microenvironment has been associated with the dysregulation of oncosuppressors or oncogenes, contributing to the development or inhibition of tumors. MiRNAs can promote or inhibit the expression of target genes by directly binding with their target mRNA. They can also affect mRNA stability [29], and aberrant miRNA regulation can make a major contribution to the development of cancer [30]. In this way, miRNAs with oncogenic function are upregulated and responsible for silencing tumor-suppressor genes that can modulate the onset, development, and metastasis of cancer cells. Conversely, miRNAs with tumor-suppressor function are downregulated, reducing the modulation of oncogenes and maintaining malignity [8].

### 2.1. Exosomal miRNAs with Diagnostic Capacity

One advantage of salivary exosomal miRNA over other OSCC biomarkers is that it can be detected in small amounts of saliva and by analysis of the whole saliva sample or the supernatant. The following salivary exosomal miRNAs have been correlated with disease stage, histopathological type and/or grade: miRNA-24-3P, 412-3p, 512-3p, 302b-3p, 517b-3p, 134, 486-5p, 4484, 10b-5p, 200a, and 365 [8,9,10,11,31,32]. Notably, the expression of miRNA-34 was greater in high-grade OSCCs, and the expression of miRNA-486-5p assisted the detection of stage I OSCC, demonstrating the potential usefulness of salivary exosomal miRNAs for the early diagnosis of cancer [10]. In the same way, it has been found that miRNAs-4484 and 10b-5p are biomarkers of the malignant transformation of oral lichen planus and oral dysplasia in OSCC [8,10,11], and a lower expression of miRNA-200a has been associated with a higher risk of malignant transformation and oral cancer. Finally, miR-365 has been found to regulate transcription, promoting oncogenesis and metastasis in some cancers but suppressing these processes in others [12].

### 2.2. MiRNAs with Tumor-Suppressor Role

MiRNAs with a tumor-suppressor role include: miRNA-27b, which inhibits cell proliferation, migration, and invasion in OSCC [14]; miRNA-200 [33], which inhibits cell growth at increased salivary concentrations [13]; miRNA-375, found to act as tumor-suppressor in multiple cancers, including OSCC [15]; and miRNA-26a, which inhibits cell migration and metastasis and lowers the expression of enhancer of zeste homolog 2 (EZH2), reducing cell growth [17]. Many tumor-suppressor miRNAs act by inhibiting the expression of genes that promote cell proliferation. These include miRNA-7 [16], which inhibits the expression of epidermal growth factor receptor (EGFR), and miRNA-107, which inhibits Akt, Stat3, and Rho GTPases genes through protein kinase Cε (PKCε) [18]. Other tumor-suppressor miRNAs inhibit cell migration, invasion, and metastasis by interfering with signaling cascades, including: miR-218 [19], which inhibits the focal adhesion pathway, impeding cell migration; some members of the miRNA let-7 family [20]; and miRNA-125 [34].

### 2.3. MiRNAs with Tumor-Activator Role

All of the following miRNAs are detected at higher concentrations in the saliva of patients with OSCC versus healthy individuals, offering a reliable method to detect OSCC and potentially malignant oral lesions [21,22,23,24].

MiRNA-21 acts as an oncogene, promoting OSCC development and progression by inhibiting apoptosis [22]. It is a marker of malignant transformation, indicating a worse prognosis and reduced survival [1]. Aberrant expression of MiR-145 has also been found to indicate malignant transformation. MiRNA-93 has been associated with OSCC, showing elevated expression at 12 months post-radiotherapy [23]. This miRNA can be considered a valuable marker to predict the prognosis and risk of metastasis, given the significant relationship found between its high expression and T grade, lymph node metastases, and clinical stage [25]. For its part, miRNA-184 is the only miRNA to date that can differentiate between OSCC and premalignant dysplastic diseases [22]. MiRNA-31 has been reported to increase the proliferation, migration, and growth of OSCC cells in vitro and in mice models, increasing their oncogenic potential [26]. It does not differentiate among tumor stages but is a good indicator of the presence of lymphatic metastasis. MiR-412-3p is positively regulated and induces cancer progression via the transforming growth factor (TGF) pathway [25]. Finally, miRNA-34a is downregulated and differentially expressed in patients with leukoplakia versus healthy individuals and has been significantly associated with the locoregional aggressiveness of tumors and their histopathological grade [22].

### 2.4. Circular RNA in OSCC Diagnosis

It has recently been demonstrated that circular RNAs (circRNAs) can serve as potential molecular markers for the diagnosis of diseases, but few data have been published on their diagnostic potential for OSCC. Zhao et al. [35] observed that circRNAs hsa_circ_0001874, hsa_circ_0001971, and hsa_circ_0008068 were upregulated and hsa_circ_0000140, hsa_circ_0002632, and hsa_circ_0008792 were downregulated in patients with OSCC versus healthy individuals. Salivary hsa_circ_0001874 was correlated with TNM stage (*p* = 0.006) and tumor grade (*p* = 0.023) and hsa_circ_0001971 with TNM stage (*p* = 0.019), and salivary expressions of hsa_circ_0001874 and hsa_circ_0001971 were found to be lower in post-operative versus pre-operative samples (*p* < 0.001).

## 3. Salivary Cytokines in OSCC Diagnosis and Prognosis

Inflammatory processes are crucial in protecting against aggressions from external infectious agents, traumatic-type tissue injuries and tumor processes. During the process by which the body recognizes these different aggressions, the production of a wide variety of inflammatory and anti-inflammatory mediators is triggered, including cytokines [36].

Cytokines are proteins secreted by certain cell groups (mainly macrophages and helper T cells), with a very important role in immunomodulation processes. Dysregulation in the levels of certain cytokines is related to the appearance of various types of cancer, considering an important role as biomarkers for the diagnosis and monitoring of certain tumor processes [37] (Table 2).

### 3.1. IL-6

Interleukin-6 (IL-6) is an important proinflammatory cytokine produced by epithelial cells, mast cells, and hematopoietic line cells, among others. It plays a role in multiple organs and systems and has a major influence at immune level, being essential for host protection during the initial stages of infection; in addition, its concentration is elevated in inflammatory diseases [66,67,68,69,70].

Elevated IL-6 concentrations have been reported in the saliva of patients with OSCC [41,42,51,53,54], being related to the inflammatory process produced by the disease [71]. They have been detected at initial stages of OSCC [38,49] and in premalignant lesions related to its onset [47]. IL-6, alongside tumor necrosis factor alpha (TNF-α), was found to discriminate between OSCC and oral leukoplakia [38]. This cytokine, among other proinflammatory proteins, has been reported to promote tumor growth and invasion, epithelial-mesenchymal transition, and angiogenesis in patients with OSCC, increasing the immune resistance of the tumor [36,38,72]. Elevated IL-6 concentrations have been associated with greater aggressiveness and severity of the disease, reducing survival and increasing the recurrence rate [48,73,74]. Concentrations of IL-6 and TNF-α were both found to increase exponentially with the progression of OSCC [38], confirming the involvement of certain proinflammatory cytokines in this disease, promoting the survival and proliferation of malignant cells [51].

### 3.2. IL-8

Interleukin-8 (IL-8) is a chemotactic factor that plays a key role in inflammatory and angiogenesis processes [75] and has important immune functions [76]. High IL-8 expression has been detected in carcinogenic cells and tissues and in the peripheral blood of patients with cancer [75,77].

Numerous authors have described elevated salivary IL-8 concentrations in patients diagnosed with OSCC [38,43,53]. In common with IL-6, it is responsible for the growth and proliferation of tumor cells and for enhancing their immune escape mechanisms [78,79]. Sahibzada et al. associated IL-8 with the aggressiveness of OSCC and supported the diagnostic validity of serum and saliva concentrations of this interleukin, even for an early detection of the disease [80]. Elevated IL-8 concentrations have been described in patients with premalignant lesions such as lichen planus, oral leukoplakia, and oral submucosal fibrosis [47], and elevated concentrations of IL-6 and IL-8 in saliva and serum have been associated with reduced survival and an increased recurrence rate in OSCC [48].

### 3.3. TNF-α

TNF-α is a transmembrane protein with a central role in triggering inflammatory reactions of the innate immune system. Its production is induced by bacterial pathogens and other microorganisms, triggering a highly complex biological cascade that involves the production of multiple anti-inflammatory biomolecules [33,81]. Elevated concentrations have been found in cancer patients at the tumor site and in the blood as a tumor survival mechanism [82,83]. As in the case of IL-6 and IL-8, this proinflammatory molecule is implicated in the growth, proliferation, and immune escape of tumor cells [78].

Its presence in saliva is elevated in patients with OSCC and is even detected at initial stages of the disease, increasing with disease progression and permitting differentiation between OSCC and oral leukoplakia [38]. Elevated concentrations of this and other biomolecules have also been found in premalignant lesions associated with progression to OSCC. Krishnan et al. [50] reported that TNF-α was highly overexpressed in patients with stage IV OSCC in comparison to patients with stages I, II, or IIIF, supporting its relationship with advanced stages of the disease.

### 3.4. MMP-9

Matrix metallopeptidase 9 (MMP-9), a protein of the endopeptidase family, degrades proteins of the extracellular matrix and participates in its remodeling in different physiological and pathological processes. This degradation of the extracellular matrix plays an important role in tumor invasion and metastasis, and MMP-9 overexpression has been used by some authors to distinguish among different types of cancer [84,85].

The presence of MMP-9 in saliva has been associated with OSCC, and its study has been proposed as a useful non-invasive approach to obtain an early diagnosis [44,45]. Shin et al. [39] reported that lower MMP-9 concentrations in patients with OSCC after than before surgery also indicate the possible prognostic value of this biomarker.

### 3.5. IL-1-β

Interleukin-1-β (IL-1-β), which acts as mediator of the immune response, is well known for its pyrogenic properties and its capacity to activate lymphocytes, stimulating their proliferation and differentiation. Its production is mediated by an inflammasome complex, and it plays a major role in the activation of various intracellular signaling cascades. IL-1-β secretion promotes numerous metabolic, physiological, and inflammatory effects, and an excess of this cytokine can produce tissue damage associated with multiple inflammatory and autoimmune diseases [86,87,88,89].

IL-1-β concentrations in both stimulated and non-stimulated saliva samples are markedly higher in patients with OSCC than in healthy patients [90]. Li et al. [40] found an elevated expression of this cytokine not only in saliva but also in neoplastic tissue from patients, especially in the initial stages of OSCC, indicating the potential diagnostic value of this biomarker. IL-1-β has been found to have diagnostic value across populations with different ethnicities. However, it is not pathognomonic, because concentrations can be increased in patients with other inflammatory diseases of the oral cavity, including periodontitis; hence, concentrations of other biomarkers must also be considered to establish a diagnosis [56]. Kamatani et al. [57] reported a decrease in salivary IL-1β after the surgical resection of OSCC, supporting the diagnostic usefulness of this cytokine to detect disease recurrence.

### 3.6. IL-1-Ra

IL-1 receptor antagonist (IL-1-Ra) binds to the membrane cell receptor of IL-1 without producing an intracellular effect, impeding the binding of IL-1 and acting as its negative regulator. IL-1-Ra is mainly produced by activated monocytes, macrophages, neutrophils, and fibroblasts after their stimulation by lipopolysaccharide, triggering a cascade of proinflammatory cytokines (e.g., IL-1 or TNF) in a first phase and a cascade of inflammation mediators (e.g., IL-10 or IL-1-Ra) in a second. IL-1-Ra has a much lower affinity for the receptor in comparison to IL-1 and needs higher concentrations to fulfill its function [58].

Niklander et al. [58] found that IL-1-Ra is constitutively expressed in normal oral epithelium but shows a reduced expression in neoplastic tissue. Its expression decreases in both primary cultures and dysplastic cells with senescence, consistent with the higher frequency of OSCC among people over the age of 50 years [91]. IL-1-Ra overexpression has been detected in dysplastic and neoplastic cells and may, alongside the overexpression of IL-1 β, favor cancer growth by regulating CD184 expression. Shiiba et al. [59] reported that IL-1-Ra had a sensitivity of 70% and specificity of 85% to discriminate between OSCC, in which it is upregulated, and other potentially malignant oral cavity diseases such as lichen planus. However, inadequate data are available on variations in its concentration over the course of OSCC, reducing its value as a prognostic marker [60,92].

### 3.7. IL-10

IL-10 is an anti-inflammatory cytokine that inhibits the synthesis of proinflammatory cytokines (e.g., IFN-γ, IL-2, IL-3, or TNF-α) by T lymphocytes and macrophages and interferes with the activity of antigen-presenting cells [93].

Elevated concentrations of this cytokine have been found in patients with OSCC [60,94,95], and very high concentrations have been associated with aggressive phenotypes of this disease, implying a worse prognosis. Suppression by IL-10 of antitumor immunity mechanisms (preventing antigen presentation from transformed cancer cells to cytotoxic T cells) favors propagation of the cancer and reduces patient survival [94,95]; IL-10 can therefore serve as a prognostic biomarker, especially at early stages of the disease. IL-10 production is very high at advanced stages due to its abundant expression by metastatic tissues, maintaining an environment that favors the proliferation and expansion of neoplastic cells that in turn segregate IL-10, in a vicious cycle [61].

### 3.8. 8-OHdG (8-Oxo-dG)

8-Oxo-2′-deoxyguanosine (8-OHdG) is a derivative of deoxyguanosine and one of the main products of DNA oxidization. It is a direct biomarker of cellular oxidative stress, which activates the expression of inflammatory genes through an enzymatic cascade, thereby contributing to carcinogenesis. Hence, 8-OHdG8 acts via a dual mechanism: gene expression and gene damage mutations by oxidization. Although it may be less well known in comparison to interleukins, 8-OHdG has demonstrated very high diagnostic value, with numerous studies reporting a strong correlation between its concentration and the presence of cancer [64,96,97,98,99].

Salivary concentrations of this biomarker were found to significantly differ among healthy individuals, patients with premalignant diseases of the oral cavity, and patients with OSCC, supporting its diagnostic and prognostic value. In the same line, Kumar et al. [64] observed that 8-OHdG, alongside reactive oxygen species and nitrogen, is more abundant in saliva from patients with head and neck squamous cell carcinoma in comparison to healthy individuals and that glutathione concentrations and total antioxidant capacity are significantly lower. However, other salivary biomarkers must be taken into consideration to establish a diagnosis, because elevated concentrations have also been detected in patients with periodontitis, who show much higher concentrations of 8-OHdG in comparison to other markers of oxidative stress, although they can be normalized by the administration of anti-inflammatories [100].

## 4. Conclusions

Accumulated evidence indicates that the measurement of miRNAs and certain oral cytokines in saliva is a highly promising technique for the diagnosis and prognosis of OSCC. A combination of salivary miRNA and proteomic data could allow a definitive and early diagnosis to be obtained. The analysis of these salivary biomarkers together with the study of other histopathological markers such as the presence of eosinophils and the immune phenotype could be a key factor in developing new strategies in OSCC treatment [101,102]. The main limitation of this review is that the included studies show high heterogeneity with respect to the methods and protocols used for miRNA and cytokines analysis. In addition, the cohort of patients in some of these studies is small. Therefore, there remains a need to optimize and standardize these protocols and design new studies with larger patient cohorts.

## Figures and Tables

**Table 1:** Salivary microRNA related to diagnosis and prognosis of oral squamous cell cancer.

Reference	Biomarker	Findings	Clinical Relevance
[8,9,10,11,12]	miRNA-24- miRNA 3P miRNA412-3p miRNA 512-3p miRNA302b-3p miRNA 517b-3p miRNA 134 miRNA 486-5p miRNA 4484 miRNA 10b-5p miRNA 200a miRNA 365	Correlated with disease stage, histopathological type, and/or grade of OSCC	Possible tool for the early diagnosis of OSCC; in general, the expression of these miRNAs indicates poor prognosis and higher risk of malignant transformation and oral cancer
[13,14,15,16,17,18,19,20]	miRNA-27b miRNA-200 miRNA-375 miRNA-26a miRNA-7 miRNA-107 miRNA-218 miRNA let-7 miRNA-125	Tumor-suppressor role	Increased levels of these biomarkers can be useful for the diagnosis, staging, and prognosis of OSCC; the expression of these miRNAs reduce the progession of OSCC
[1,21,22,23,24,25,26]	miRNA-21 miRNA-145 miRNA-93 miRNA-184 miRNA-31 miRNA-412-3p miRNA-34a	Tumor-activator role; these miRNAs acts as an oncogenes, promoting OSCC development and progression	Elevated concentrations in the saliva offer a reliable method to detect OSCC and potentially malignant oral lesions

**Table 2:** Salivary biomarkers related to diagnosis and prognosis of oral squamous cell cancer.

Reference	Biomarker	Findings	Clinical Relevance
[38]	IL-6, IL-8 & TNF-α	Notably higher levels of these cytokines in advanced stages of OSCC compared to early stages of the disease; presence of neck metastases associated with increased levels of these molecules.	Possible tool to indicate OSCC progression
[39]	MMP-9	Elevated salivary levels of MMP-9 were associated with OSCC Levels of the biomarker decreased dramatically after tumor surgery	MMP-9 as a critical diagnostic and prognostic biomarker for OSCC
[40]	IL-6, IL-8, IL-1β & TNF-α	Significant differences in levels of IL-6, IL-8, IL-1β, and TNF-α between OSCC patients and to controls	Useful complementary tool for the early detection of OSCC
[41]	IL-8	Significantly increased levels of the cytokine in patients with head and neck squamous cell carcinoma; IL-8 levels were positively correlated with the abundance of *C. albicans*	A salivary microbial and inflammatory biomarker of head and neck squamous cell carcinoma that is influenced by oral health
[42]	IL-6 & IL-8	Correlation of qualitative salivary detection of IL-6 and IL-8 between control and disease groups	Probable biomarker for detection of premalignant lesions and OSCC
[43]	IL-6 & TNF-α	Elevated levels of those cytokines compared to age-matched controls	IL-6 and TNF-α are potential biomarkers for the monitorization of OSCC
[44]	MMP-9	MMP-9 levels significantly higher in OSCC patients than in controls or patients with premalignant lesions	Salivary diagnostic biomarker for the detection of premalignant oral lesions and early stages of OSCC
[45]	MMP-9	Higher levels of MMP-9 in OSCC patients than in controls	MMP-9 is a good tool for the detection of OSCC
[46]	IL-8	Protein concentration of IL-8 was significantly elevated in patients with OSCC than in those with dysplasia and controls	Important marker to discriminate between OSCC and control patients; IL-8 combined with H3F3A mRNA provides good discrimination between OSCC and potentially malignant oral disorders
[47]	IL-6, IL-8 & TNF-α	Increased levels of these cytokines in patients with oral leukoplakia, submucous fibrosis, and lichen planus than in healthy controls	Diagnostic tool for the detection of premalignant lesions
[48]	IL-6	Higher pretreatment levels of IL-6 in patients with oral cancer, associated with better survival	Possible prognosis biomarker
[49]	IL-6	Higher salivary levels of IL-6 in OSCC patients when compared with patients with chronic periodontitis, active oral lichen planus, inactive oral lichen planus, or healthy controls	Useful biomarker for the detection of OSCC
[50]	TNF-α	Increased serum and saliva TNF-α levels in OSCC patients compared with controls and those with premalignant disease	TNF-α as a useful biomarker for OSCC detection; increased levels are associated with histological grade and clinical stage, suggesting a role in the prognosis of OSCC
[51]	IL-6	Increased levels	Monitoring of OSCC
[48]	TNF-α	No differences between control and OSCC group	-
[52]	IL-6, IL-8 & TNF-α	Higher levels in endophytic squamous cell carcinoma of the tongue than in exophytic squamous cell carcinoma of the tongue, correlated with decreased survival in the endophytic versus exophytic group; IL-6, IL-8, and TNF-α also higher in the exophytic group than in smoking and drinking controls	These biomarkers can identify the progression of squamous cell carcinoma of the tongue from high risk to neoplasm; important biomarker for cancer screening and early detection; correlation between these proteins and survival implies a prognostic benefit potentially useful for management decisions and future target treatments
[53]	IL-6 & IL-8	Higher expression in patients with OSCC	Potential tool for OSCC diagnosis
[54]	IL-6, IL-8 & TNF-α	Increased levels in patients with OSCC and premalignant oral lesions	Proangiogenic and proinflammatory cytokines are elevated in patients with these lesions; diagnosis and prognosis significance of these markers
[55]	IL-8 & IL-1β	Increased levels in patients with OSCC	Potential use as a diagnostic tool for OSCC
[56]	IL-8 & IL-1β	Higher levels in OSCC patients, depending on the tumor stage	Increased levels of these biomarkers can be useful for OSCC diagnosis, staging, and prognosis
[57]	IL-1β	Levels significantly differ between before and after surgery	IL-1β levels may be useful for the detection of early stage OSCC
[58]	IL-1-Ra	Expression of IL-1-Ra is lower in OSCC and oral dysplasia cells than in normal cells	Possible use as a biomarker for prediction of malignant transformation
[59]	IL-1-Ra	Expression of IL-1-Ra decreases gradually with the progression of oral dysplasia	IL-1-Ra could be a reliable biomarker for the early diagnosis and follow-up of OSCC; it could be useful to discriminate between premalignant oral lesions and OSCC
[60]	IL-1-Ra & IL-10	Salivary IL-10 levels are higher in OSCC patients; IL-1-Ra levels are lower in well-defined tumors than in immature tumors	IL-10 is an interesting tool for diagnosing OSCC, and IL-1-Ra can be helpful for cancer staging
[61]	IL-10	High levels of IL-10 expression are found in OSCC, especially in advanced stage tumors and metastatic cells	Salivary IL-10 levels could be used as a biomarker for OSCC diagnosis; a high concentration appears to favor tumor proliferation and dissemination
[62]	IL-10	High levels of IL-10 expression correlate with shorter survival, worse prognosis, and increased risk of death	Overexpression of IL-10 is associated with aggressive forms of OSCC, and its level can be used as a survival predictor
[63]	IL-10	IL-10 levels increase with tumor progression	Useful as staging biomarker
[64]	8-OHdG	8-OHdG levels are approximately two-fold higher in patients with squamous head and neck cancer than in healthy controls	Quantification of 8-OHdG levels could be used as a diagnostic tool for OSCC
[65]	8-OHdG	8-OHdG levels are more than two-fold higher in in OSCC patients than in controls	8-OHdG can be used as DNA damage biomarker to assess disease progression

## Abbreviations

OSCC	Oral squamous cell carcinoma
EVs	Extracellular vesicles
EZH2	Enhancer of zeste homolog 2
EGFR	Epidermal growth factor receptor
PKCε	Protein kinase Cε
TGF	Transforming growth factor
circRNAs	Circular RNAs
IL-6	Interleukin-6
TNF-α	Tumor necrosis factor alpha
IL-8	Interleukin-8
MMP-9	Matrix metallopeptidase 9
IL-1-β	Interleukin-1-β
IL-1-Ra	IL-1 receptor antagonist
IL-10	Interleukin-10
8-OHdG (8-oxo-dG)	8-Oxo-2′-deoxyguanosine
